# Supplementary material for: Splice-Junction-Based Mapping of Alternative Isoforms in the Human Proteome
Source: Cell Rep. Author manuscript; Available in PMC 2020 Jan 15. (PMC6961840; doi:10.1016/j.celrep.2019.11.026)
Supplement: 2 [file NIHMS1546469-supplement-2.zip › DF1/PXD010154/Thyroid/Thyroid_42_TG_ILDCGSPDIEVHTYPFGWYQKPMFK.pdf]

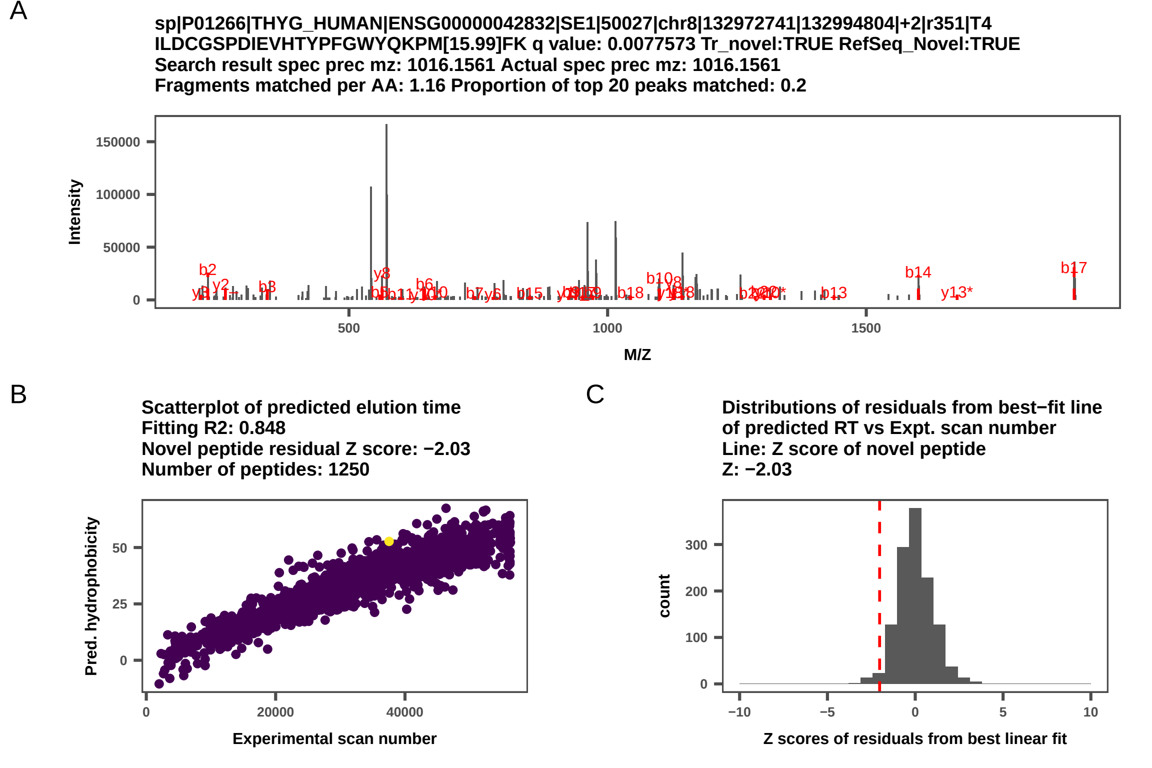
A
sp|P01266|THYG_HUMAN|ENSG00000042832|SE1|50027|chr8|132972741|132994804|+2|r351|T4
ILDCGSPDIEVHTYPFGWYQKPM[15.99]FK q value: 0.0077573 Tr_novel:TRUE RefSeq_Novel:TRUE
Search result spec prec mz: 1016.1561 Actual spec prec mz: 1016.1561
Fragments matched per AA: 1.16 Proportion of top 20 peaks matched: 0.2
Intensity
150000
100000
50000
0
b2
y2
b3
y8
b6
b14
b17
b18
b13
y13*
M/Z
500
1000
1500
B
Scatterplot of predicted elution time
Fitting R2: 0.848
Novel peptide residual Z score: −2.03
Number of peptides: 1250
Pred. hydrophobicity
50
25
0
0
20000
40000
Experimental scan number
C
Distributions of residuals from best−fit line
of predicted RT vs Expt. scan number
Line: Z score of novel peptide
Z: −2.03
count
300
200
100
0
−10
−5
0
5
10
Z scores of residuals from best linear fit
